# Safety and Effects of *Lactobacillus paracasei* TISTR 2593 Supplementation on Improving Cholesterol Metabolism and Atherosclerosis-Related Parameters in Subjects with Hypercholesterolemia: A Randomized, Double-Blind, Placebo-Controlled Clinical Trial

**DOI:** 10.3390/nu15030661

**Published:** 2023-01-28

**Authors:** Jurairat Khongrum, Pratoomporn Yingthongchai, Kongsak Boonyapranai, Wachira Wongtanasarasin, Paitoon Aobchecy, Suriya Tateing, Aree Prachansuwan, Jaruwan Sitdhipol, Kanidta Niwasabutra, Punnathon Thaveethaptaikul, Pongsathon Phapugrangkul, Pennapa Chonpathompikunlert

**Affiliations:** 1 Science and Technology Research Institute, Chiang Mai University, Chiang Mai 50200, Thailand; 2 Functional Food Research Center for Well-Being, Science and Technology Research Institute, Chiang Mai University, Chiang Mai 50200, Thailand; 3 Research Institute for Health Science, Chiang Mai University, Chiang Mai 50200, Thailand; 4 Department of Emergency Medicine, Faculty of Medicine, Chiang Mai University, Chiang Mai 50200, Thailand; 5 Department of Plant and Soil Sciences, Faculty of Agriculture, Chiang Mai University, Chiang Mai 50200, Thailand; 6 Institute of Nutrition, Mahidol University, Nakhon Pathom 73170, Thailand; 7 Biodiversity Research Centre (BRC), Thailand Institute of Scientific and Technological Research (TISTR), Pathumthani 12120, Thailand

**Keywords:** *Lactobacillus paracasei*, probiotics, hypercholesterolemia, oxidative stress, inflammation, apolipoprotein E, clinical trial

## Abstract

Probiotics have the potential as a multi-target approach to modulate hypercholesterolemia associated with premature atherosclerosis. Various strains of *Lactobacillus paracasei* have been reported to affect hypercholesterolemia positively. This study aimed to investigate the effects of *L. paracasei* TISTR 2593 on lipid profile, cholesterol metabolism, and atherosclerosis according to the registration of Thai Clinical Trial Registry as identification number TCTR 20220917002. A total of 50 participants with hypercholesterolemia were randomly and equally assigned to consume *L. paracasei* TISTR 2593 or a placebo in maltodextrin capsules daily. Biomarkers of lipid profiles, oxidative stress state, inflammatory state, and other biological indicators were examined on days 0, 45, and 90. The results showed that subjects taking the *L. paracasei* TISTR 2593 could significantly reduce the level of serum low-density lipoprotein-cholesterol (*p* < 0.05), malondialdehyde (*p* < 0.001), and tumor necrosis factor-α (*p* < 0.01). Moreover, *L. paracasei* TISTR 2593 increased the level of serum apolipoprotein E (*p* < 0.01) and adiponectin (*p* < 0.001) significantly. No changes in serum total cholesterol, high-density lipoprotein-cholesterol, triglyceride, total bile acids, and monocyte chemoattractant protein-1 were observed during *L. paracasei* TISTR 2593 supplementation. Therefore, *L. paracasei* TISTR 2593 could be an adjuvant probiotic supplement to ameliorate hypercholesterolemia and prevent or delay the development of atherosclerosis.

## 1. Introduction

Hypercholesterolemia is a major risk factor leading to cardiovascular diseases (CVDs), a major cause of death worldwide. High levels of total cholesterol, low-density lipoprotein-cholesterol (LDL-C), and triglyceride result in a build-up of fatty deposits inside the arteries leading to atherosclerosis [1]. According to the American College of Cardiology (ACC) and American Heart Association (AHA) Guideline, LDL-C lowering is a major tool in cholesterol management and primary prevention of cardiovascular disease [2]. 

Accumulating evidence shows that the elevated levels of LDL-C predominantly induce oxidative modification of LDL-C in arterial walls through enzymatic and nonenzymatic oxidation of lipid molecules, leading to aggravation of oxidative stress and inflammatory responses in the vascular endothelium cell resulting in the emergence of oxidized low-density lipoprotein (oxLDL) [3]. The elevation of OxLDL could stimulate vascular endothelial cells to secrete the chemokine monocyte chemoattractant protein 1 (MCP-1) contributing role on a foam cell formation involved in the initial stages of atherogenesis constitutes [4]. Therefore, the accumulation of LDL-C is considered a cause of atherosclerotic lesions. 

Substantial evidence from clinical and experimental studies has demonstrated that alterations in cholesterol and lipoprotein metabolism [5] such as reduced bile acid excretion [6], decreased apolipoprotein E concentration, [7] and impaired adiponectin function [8] play an important role in hypercholesterolemia related to the incidence of CVDs. Therefore, the changes as mentioned earlier have been increasingly motioned for the likelihood of discovering new cholesterol-lowering drugs, which are more effective in the prevention and therapeutic intervention of CVDs [9,10].

In recent years, several food supplements have emerged as potentially effective for dyslipidemia, lowered LDL-C levels, and CVD prevention, such as garlic [11], soy protein [12], probiotics [13], nuts [14], and cocoa/chocolate [15]. Several clinical studies have been conducted to determine the cholesterol-lowering effects of probiotic supplements in people with hypercholesterolemia [16,17]. Numerous studies on *Lactobacillus paracasei* showed a potential effect in reducing cholesterol levels and preventing atherosclerosis [18,19,20]. There are several pathways to ameliorate hypercholesterolemia with positive effects from *L. paracasei* such as antioxidant activity [21], anti-inflammation activity [18], immunomodulatory activity [22], and cholesterol metabolism modification [23]. However, the results regarding the cholesterol-lowering effect of *L. paracasei* are insufficient due to a lack of a comprehensive multi-targeted approach to disease status, and there are no clinical studies examining the cholesterol-lowering effect together with anti-oxidative stress, anti-inflammation, and improved cholesterol metabolism activity.

Therefore, the objective of this study was to investigate the effect of *L. paracasei* TISTR 2593 in a maltodextrin capsule in a randomized control trial on blood chemistry, fasting blood glucose, and serum lipid profile. In addition, this study also investigated *L. paracasei* TISTR 2593 against hypercholesterolemia-related atherosclerosis via serum levels of oxidative stress status, inflammation cytokines, adiponectin, apolipoprotein E, total bile acids, and monocyte chemoattractant protein-1 (MCP-1) in the serum of hypercholesterolemic subjects. 

## 2. Materials and Methods

### 2.1. Subjects

This study was conducted following the rules of the Declaration of Helsinki. The protocol was approved by the Ethical Committee of the Human Experimentation Committee, Research Institute for Health Science (RIHES), Chiang Mai University, Thailand (Project No. 11/64), and the Thai Clinical Trials Registry (TCTR) is TCTR20220917002 (https://www.thaiclinicaltrials.org/show/TCTR20220917002, accessed on 6 September 2022). All subjects provided informed consent before participation in the study. 

Subjects were randomly distributed into two groups: placebo or *L. paracasei* TISTR 2593. The inclusion criteria were male and female (non-pregnant), aged 30–65 years, mild and moderate hypercholesterolemia (defined by having serum LDL-C between 100 and 159 mg/dL), body mass index (BMI) between 19 and 30 kg/m^2^, no vegan, no vegetarian, and without cholesterol- and triglyceride-lowering drug consumption. In addition, subjects were excluded in the case of cardiovascular disease events and secondary dyslipidemia. Subjects were advised about lifestyle recommendations, but made no change in their exercise, eating habits, taking specific diet or other supplements during the whole study period. They were contacted twice weekly to ask about any adverse effects during the study. 

In addition, subjects in this study underwent a general characteristics examination, including age, sex, BMI, systolic blood pressure, diastolic blood pressure, and heart rate, and were interviewed using a questionnaire to obtain information on their dietary status.

### 2.2. Study Design and Treatment

We conducted a single-center, prospective, randomized, double-blind, placebo-controlled, parallel-group trial. Subjects were kept blind regarding treatments. Blinded investigators performed data interpretation and analysis.

**Figure 1:**
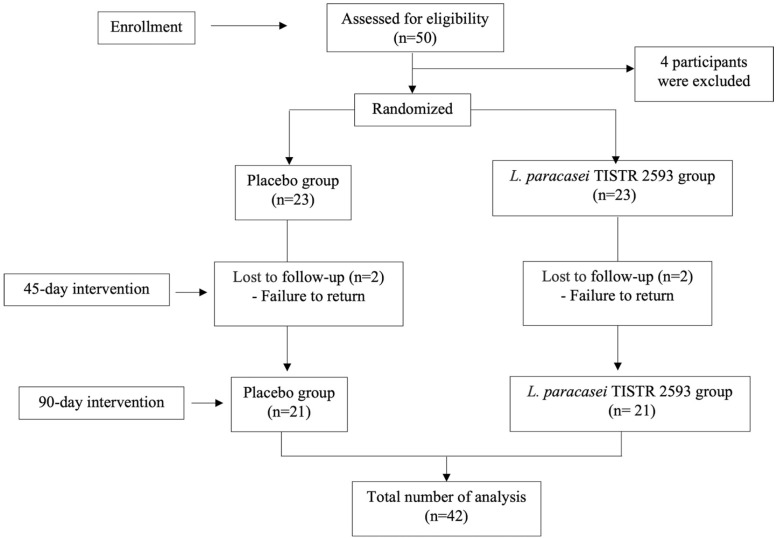
Consort flow diagram.

Subjects were randomly assigned using block randomization into two groups as follows: (1) *L. paracasei* TISTR 2593 in maltodextrin capsule obtained from the Expert Center of Innovative Health Food, Thailand Institute of Scientific and Technological Research, Thailand, at a daily dose of 1.05 × 10^10^ CFU/g (350 mg per capsule); and (2) a placebo product which was a maltodextrin capsule. In addition, subjects consumed the assigned substance once daily before breakfast. Blood was collected to determine a lipid profile consisting of serum levels of total cholesterol (TC), triglyceride (TG), LDL-C, high-density lipoprotein-cholesterol (HDL-C) together with fasting blood glucose (FBG), blood safety parameters, and other parameters at a period prior to the intervention, then at 45-day and 90-day intervention periods. The study flowchart and enrollment are shown in Figure 1.

### 2.3. Blood Sampling and Biochemical Measurements

Blood samples were collected in the morning after 12 h fasting at each visit and kept in a test tube to analyze for blood lipid profiles (TG, TC, LDL-C, and HDL-C), serum biochemistry (FBG, blood urea nitrogen (BUN), creatinine, and alanine transaminase (ALT), aspartate transaminase (AST) and alkaline phosphatase (ALP), blood electrolytes (sodium, potassium, and chloride), and complete blood count examinations as the following parameters: white blood cell count (WBC), hemoglobin concentration, hematocrit, platelet count (PLT), neutrophil, lymphocyte, monocyte, eosinophil, mean corpuscular volume (MCV), mean corpuscular hemoglobin (MCH), and mean corpuscular hemoglobin concentration (MCHC). The tests were done in the Laboratory Unit of Chiang Mai Medical Lab, Chiang Mai, Thailand. Additionally, other blood samples were allowed to clot and were spun at 1450 RCF or g force for 10 min at 4 °C. Then, the serum was transferred into dry well-labeled specimen plastic tubes and stored at −80 °C until analysis. Serum analysis was performed on a spectrophotometry (SPECTROstarNano, BMG LABTECH, Biotechnology division, Scientific Promotion Ltd., Ortenberg, Germany) analyzer to study a change of oxidative stress status, an inflammation marker, lipid metabolism enzymes, and MCP-1 parameter. 

The oxidative stress and antioxidant markers were used to evaluate the oxidative stress status. The lipid peroxidation level, excess production of reactive oxygen species (ROS), was investigated using the thiobarbituric acid reactive substances (TBARS) method according to Bhutia et al. [24], and glutathione (GSH) level, which is an antioxidant capacity to mitigate oxidative stress, was determined according to the previous study [25]. 

Inflammatory markers, including interleukin-10 (IL-10), interleukin-6 (IL-6), and tumor necrosis factor-α (TNF-α), were measured by enzyme-linked immunosorbent assay kits using paired antibodies (Sigma^®^, Burlington, MA, USA for IL-10; Abcam^®^, Waltham, MA, USA for IL-16; and Elabscience^®^, Houston, TX, USA for TNF-α). The minimum detectable dose of IL-10, IL-6, and TNF-α is typically less than 1.4 pg/mL, 1.6 pg/mL, and 4.69 pg/mL, respectively. The intra- and interassay coefficient of variations (CVs) of IL-10, IL-6, and TNF-α were <10%, <5%, and <10%, respectively.

The levels of total bile acid (TBA), adiponectin, apolipoprotein E (APOE), and monocyte chemoattractant protein-1 (MCP-1) were also evaluated via enzyme-linked immunosorbent assay kits with the intra- and interassay coefficient of variations (CVs) <12%, <10%, <10%, and <10%, respectively. (Elabscience^®^, Houston, TX, USA for TBA; Abcam^®^, Waltham, MA, USA for adiponectin; and Elabscience^®^, Houston, TX, USA for Apo E and MCP-1). The minimum detectable dose of TBA, adiponectin, APOE, and MCP-1 is typically less than 2.05 μmol/L, 0.18 ng/mL, 14.06 ng/mL, and 37.50 pg/mL, respectively.

### 2.4. Statistical Analysis

The sample size of fifty subjects was calculated using the superiority design method (calculating sample size when the outcome measure is a continuous variable) [26] based on the mean difference of LDL-C reductions with a significance level of 0.05 [27]. Twenty-five subjects were enrolled in each group. A total of fifty patients (twenty-five subjects in each group) was calculated to achieve a statistical power of 80%, giving a 10% dropout rate).

Statistical analysis was performed using SPSS software version 22 (SPSS Inc., Chicago, IL, USA). Data were ensured using the normal distribution of variables by the Kolmogorov–Smirnov test, and variables that were non-normally distributed were log-transformed before statistical analysis.

The descriptive analysis was expressed as an absolute number and percentage. Continuous variables were expressed using means and standard deviations (SDs) and associated two-tailed 95% confidence intervals (CIs). We applied the means substitution method to handle missing values. The sensitivity analysis in which the missing values were not imputed was performed. Outcomes were compared to the baseline during a study period, and a paired *t*-test was used to identify a significant difference. The differences between the group’s outcomes were analyzed using repeated measure ANOVA followed by an LSD *post hoc* test. The statistical significance was considered when the *p*-value was less than 0.05. 

## 3. Results

### 3.1. Characteristics of the Subjects

A total of 50 hypercholesterolemic subjects were assessed for eligibility, and 4 subjects were excluded due to the presence of secondary dyslipidemia (n = 3) and relocation (n = 1). 

Subjects were randomly allocated into the *L. paracasei* TISTR 2593 group (n = 23) and the placebo group (n = 23). Four subjects dropped out: two from the placebo group and two from the *L. paracasei* TISTR 2593 group. Finally, 42 subjects remained and were included in the analysis (Figure 1).

Subjects in the *L. paracasei* TISTR 2593 group were 48.5 ± 5.3 years old and 65.2% female whereas subjects in the placebo group were 46.0 ± 5.1 years old and 82.6% female. All subjects reported no serious adverse effects or clinical symptoms throughout the study.

The within-group and between-group analysis showed that general characteristics of BMI, systolic blood pressure, diastolic blood pressure, heart rate, and calorie intake had no statistically significant difference at baseline and the end of the study. The general characteristics data of all subjects at baseline and the end of the study were presented in Table 1.

### 3.2. Effect of L. paracasei TISTR 2593 Supplementation on Blood Safety Parameters 

Blood samples were tested for FBG, BUN, creatinine, ALT, AST, ALP, blood electrolytes, and hematology representing safety parameters when repetitively consuming *L. paracasei* TISTR 2593 supplementation for 90 days. 

A comparison of within-group and between-group was analyzed, and results are shown in Table 2. FBG, BUN, creatinine, ALT, AST, ALP, blood electrolytes, and completed blood count showed no significant difference when comparing within-group and between-group throughout the intervention.

### 3.3. Effect of L. paracasei TISTR 2593 Supplementation on Blood Lipid Profiles

To investigate the effect of *L. paracasei* TISTR 2593 on blood lipid profiles, TC, TG, LDL-C, and HDL-C were tested in hypercholesteremic subjects. The results are shown in Table 3. Consumption of a 1.05 mg/day x 10^10^ of *L. paracasei* TISTR 2593 for 45 and 90 days showed significant (*p* < 0.05 and *p* < 0.05, respectively) reduction of LDL-C level together with the mean difference from baseline at −17.75 mg/dL (95% CI: −23.84 to 18.94) and −17.52 mg/dL (95% CI: −41.59 to 1.19), respectively. In addition, a significant reduction was found in the serum level of LDL-C compared to the placebo group (*p* < 0.01). However, TC, TG, and HDL-C showed no significant differences when compared within and between groups.

### 3.4. Effect of L. paracasei TISTR 2593 Supplement on Oxidative Stress and Inflammation

Hypercholesterolemia alters vascular endothelial cell membranes, enhancing oxidative stress and inflammation mediators implicated in the pathogenesis of atherosclerosis [28,29]. The oxidative stress status including lipid peroxidation product (MDA) levels, antioxidant activity (GSH), and inflammatory parameters including IL-10, IL-6, TNF-α, and MCP-1 were also investigated to observe the possible beneficial effects of *L. paracasei* TISTR 2593.

In current results, it was found that subjects who consumed *L. paracasei* TISTR 2593 for 45 days and 90 days significantly decreased the MDA level (*p* < 0.05 and *p* < 0.001, respectively) and the mean difference from baseline was −0.03 nmol/mL (95% CI: 0.01 to 0.04) and −0.04 nmol/mL (95% CI: 0.02 to 0.06), respectively. The inflammatory parameter TNF-α level was significantly decreased (*p* < 0.01) after consuming *L. paracasei* TISTR 2593 for 90 days, and the mean difference from baseline was −5.10 pg/mL (95% CI: 1.90 to 8.30).

Additionally, a significant difference in the reduction in MDA (*p* < 0.001) and TNF-α (*p* < 0.05) levels was also found when compared to the placebo group. However, GSH, IL-10, IL-6, and MCP-1 levels did not change when compared within and between groups. The results are shown in Table 4.

### 3.5. Effect of L. paracasei TISTR 2593 Supplement on Adiponectin, apolipoprotein E, and Total Bile acid Level

Regulating glucose and lipid metabolism, cholesterol transportation, and cholesterol execration play an important role in disrupting cholesterol metabolism. Thus, the serum level of adiponectin, apolipoprotein E, and TBA was evaluated to observe the possible effect of *L. paracasei* TISTR 2593 on cholesterol metabolism protein in hypercholesteremic subjects.

Interestingly, the results showed that subjects who consumed *L. paracasei* TISTR 2593 decreased serum adiponectin levels significantly after intaking for 45 and 90 days (*p* < 0.001 and *p* < 0.001, respectively), and the changes from baseline at 45 and 90 days were −2.07 ng/mL (95% CI: 1.45 to 2.68) and −3.95 ng/mL (95% CI: 3.33 to 4.56). Similarly, the serum adiponectin level of subjects in the placebo group significantly decreased after intaking for 45 and 90 days (*p* < 0.001 and *p* < 0.001, respectively) together with the changes in baseline at −4.89 ng/mL (95% CI: 1.36 to 2.43) and −5.76 ng/mL (95% CI: 5.20 to 6.31). In addition, the serum apolipoprotein E level of subjects who consumed *L. paracasei* TISTR 2593 significantly increased throughout the study period (*p* < 0.05 at 45 days and *p* < 0.01 at 90 days), and the change from baseline was 0.12 ng/mL (95% CI: −0.21 to −0.03) and 0.14 ng/mL (95% CI: −0.23 to −0.04).

When compared between groups, we found that the placebo group’s serum adiponectin level decreased significantly than the *L. paracasei* TISTR 2593 group (*p* < 0.01), and serum apolipoprotein E did not have a significant difference. However, serum TBA levels showed no significant change after consuming either *L. paracasei* TISTR 2593 or a placebo. The results are shown in Table 4.

## 4. Discussion

This present study demonstrated the benefit of *L. paracasei* TISTR 2593 on lowering LDL-C, anti-oxidative stress, and anti-inflammation with improved cholesterol metabolism activity. In addition, daily consumption of the *L. paracasei* TISTR 2593 supplement has no adverse effect or clinical symptoms. Furthermore, data from the blood chemistry, electrolytes, and hematological values showed no significant toxicity. Therefore, these data indicated that daily consumption of *L. paracasei* TISTR 2593 (1.05 × 10^10^ CFU/g) is safe for hypercholesterolemia individuals.

Recently, it has been suggested that the gut microbiota and their metabolites play an important role in regulating lipid metabolism and glucose homeostasis [30]. A growing number of studies have shown that probiotics exert beneficial health effects by interacting with the host’s gut microbial community and resulting in changes in the production of metabolic compounds [31]. Consumption of the probiotic *Lactobacillus* strains has significantly been demonstrated to reduce serum TC and LDL-C [32]. Our results from 45- and 90-day, double-blind, placebo-controlled trials found that supplementation with the probiotic *L. paracasei* TISTR 2593 (1.05 × 10^10^ CFU/g) can significantly reduce serum LDL-C levels by 11.30% and 15.56% when compared with the placebo group in Thai adults with hypercholesterolemia without changes in diet or lifestyle. These findings emphasized that *L. paracasei* TISTR 2593 could be an alternative option in managing serum LDL-C, thereby reducing the risk of CVDs in people with hypercholesterolemia.

This observation may be attributed to the anti-inflammatory effects of probiotics by inhibiting the release of pro-inflammatory cytokines. The reduced inflammatory state in the body could be a potential metabolic process for the LDL-C lowering effect. A significant reduction in serum TNF-α without any changes in IL-6 was observed in participants receiving *L. paracasei* TISTR 2593 supplementation for 90 days. These results were consistent with a meta-analysis of Naseri et al. [33], which demonstrated that probiotic or synbiotic supplementation was associated with a decrease in TNF-α but not in IL-6 in patients with prediabetes and type 2 diabetes. In addition, Vincenzi et al. [34] reviewed that probiotic *L. reuteri* can inhibit TNF-α expression through several pathways including the inhibition of mitogen-activated protein kinase (MAPK) and the nuclear factor-kB (NF-κB) signaling pathway, the inhibition of IκB phosphorylation and nuclear translocation of NF-κB, the inhibition of signal transducer and activator of transcription-3 (STAT-3) activation, increase in negative toll-like receptor (TLR) regulators, and decrease the binding of lipopolysaccharides (LPS) to the cluster of differentiation 14 (CD14) receptor. In a mouse model, TNF-α interferes with the cholesterol metabolic pathway by increasing hepatic cholesterol synthesis through the induction of 3-hydroxy-3-methylglutaryl-CoA (HMG-CoA) reductase activity, resulting in increased circulating cholesterol levels [35]. In patients with psoriatic arthritis, the effect of anti-TNF-α therapy on TC, TG, and LDL-C was significantly reduced after 36 months of treatment [36]. Hence, this observation proposed the effect of the probiotic *L. paracasei* TISTR 2593 on LDL-C reduction through inhibition of TNF-α expression.

Our results showed that plasma APOE levels were significantly increased in participants with *L. paracasei* TISTR 2593 supplementation, but there was no significant change in the placebo group. It has been widely accepted that APOE plays a central role in the clearance of VLDL and LDL in plasma. Due to its better affinity for LDL receptors than apoB-100, APOE can promote the clearance of VLDL and LDL from plasma, reducing ischemic heart disease risk [37]. This result may be relevant to reducing plasma LDL-C and TNF-α. Some studies affirmed that proinflammatory cytokines, such as TNF-α and interferon-γ (IFN-γ), can downregulate the production of APOE [38,39]. In contrast, anti-inflammatory stimulants can promote the synthesis and release of APOE [40]. As found in this study, a reduction of TNF-α could lead to the upregulation of APOE expression.

According to the emerging evidence, it has been suggested that elevated oxidative stress is involved with endothelial dysfunction induced by hypercholesterolemia [41]. Malondialdehyde (MDA) is a lipid peroxidation product and a biomarker of oxidative stress [42]. Csonka et al. [43] showed that serum MDA and protein carbonyl (PCO) levels were positively correlated with LDL-C and TC in subjects with familial hypercholesterolemia, which is related to an increased risk of developing atherosclerosis. Our results demonstrated that serum MDA levels were significantly reduced in participants who obtained *L. paracasei* TISTR 2593 supplementation. This current finding was consistent with a systematic review and meta-analysis of 26 randomized controlled trials confirming that probiotic/synbiotic supplementation can significantly reduce serum MDA levels in adults [44]. For example, consuming a synbiotic capsule containing 4 × 10^8^ CFU *L. casei* and 400 mg of inulin for 7 weeks resulted in a significant decrease in MDA and increase in the concentrations of GSH [45]. Similarly, reductions in serum MDA were seen only in hypercholesterolemic subjects who received *L. paracasei* HII01 supplementation for 12 weeks but not in the placebo group [18]. These may be explained by the probiotic strains’ hydroxyl radical and superoxide anion scavenger properties and their antioxidant production [46]. Probiotic supplementation could inhibit lipid oxidation, thereby reducing the risk of atherosclerosis.

Adiponectin plays an important role in glucose and lipid metabolism, and adiponectin deficiency is related to insulin resistance, inflammation, dyslipidemia, and the risk of atherosclerosis [47]. However, the effect of probiotics on plasma adiponectin in humans is still inconclusive. In the present study, serum adiponectin was significantly reduced in both the probiotic and placebo groups, but the probiotic group decreased less than the placebo group. In contrast, the previous trial showed that serum adiponectin was significantly increased in obese adults given the probiotic *Lactobacillus gasseri* SBT2055 [48]. However, meta-analysis studies have found that the administration of probiotics and synbiotics did not alter plasma adiponectin and leptin levels compared with a control group [33,49]. These findings may be due to population differences, probiotic strains, probiotic dose, and consumption duration.

In addition to the above remarks, bile salt hydrolase (BSH) enzyme activity was also increased with probiotic supplementation. Co-precipitation between intestinal cholesterol and deconjugated bile salts could reduce the absorption of cholesterol and bile salts, consequently lowering blood cholesterol [50]. Nonetheless, our study observed increased total bile acids in plasma in both groups, but this was not significant. A double-blind, randomized, placebo-controlled crossover study found that *Bifidobacterium animalis* subsp. *lactis* B94 or *Bacillus subtilis* R0179 supplementation for six weeks increased plasma deconjugated bile acids in subjects with a BMI ≥30 kg/m^2^, while there was no effect of *Lactobacillus plantarum* HA-119 on plasma bile acids [51]. It seems that some strains of probiotics can modulate plasma bile acid profiles, leading to the clinical benefits mentioned earlier. To understand the effect of probiotics on bile acid metabolism, plasma concentrations of deconjugated bile salts are required for further investigation.

Even though there were no differences in baseline characteristics between groups, some limitations were still found in the study. Our study did not analyze changes in the gut microbiota community in feces, which is an essential indicator to confirm the colonization of the probiotic *L. paracasei* TISTR 2593. In addition, the participants’ diet, lifestyle, mood, and physical activity could affect or modulate the underlying mechanisms because the study was conducted in a free-living setting; however, we asked them to maintain their diet and exercise patterns during the study period and ensured by measuring calorie intake at the start and the end of the study which showed no significant changes.

## 5. Conclusions

In summary, the current study findings demonstrated that supplementation with *L. paracasei* TISTR 2593 capsules for 90 days has a lowering effect on LDL-C, anti-oxidative stress, and anti-inflammation in Thai adults with high cholesterol levels. Thus, *L. paracasei* TISTR 2593 could be an adjuvant probiotic supplement to help manage LDL-C levels and potentially delay the development of atherosclerosis. However, the underlying mechanism of how *L. paracasei* TISTR 2593 exerts the reduction in blood lipids and prevents the development of atherosclerosis, such as the connection with the gut microbiota community, should be performed in further studies which may support probiotic-based food supplementations for managing hypercholesterolemia associated with cardiovascular diseases.

## Figures and Tables

**Table 1:** The general characteristics of the subjects. Data were presented as ± standard deviations.

Parameters	*L. paracasei* TISTR 2593		*p*-Value within Group	Placebo		*p*-Value within Group	*p*-Value between Groups
	Baseline	90-Day		Baseline	90-Day		
Body mass index (kg/m^2^)	26.21 ± 3.82	25.31 ± 3.33	0.709	25.11 ± 4.24	25.79 ± 6.10	0.898	0.750
Systolic blood pressure (mmHg)	124.43 ± 19.19	123.14 ± 19.18	0.284	117.39 ± 29.19	125.95 ± 23.42	0.368	0.665
Diastolic blood pressure (mmHg)	78.43 ± 11.65	77.77 ± 10.67	0.771	80.61 ± 15.13	79.95 ± 13.55	0.987	0.967
Heart rate (Bmp)	78.43 ± 14.45	75.91 ± 11.73	0.787	81.78 ± 13.21	79.55 ± 11.28	0.737	0.785
Calorie intake (Kcal)	2771.38 ± 476.77	2558.12 ± 698.80	0.976	2670.85 ± 533.06	2645.38 ± 792.61	0.992	0.967

**Table 2:** Effect of *L. paracasei* TISTR 2593 supplementation on blood safety parameters.

Blood Parameters	*L. paracasei* TISTR 2593			*p*-Value within Group	Placebo			*p*-Value within Group	*p*-Value between Groups
	Baseline Mean ± SD	45-Day Mean ± SD Mean Difference (95% CI) from Baseline	90-Day Mean ± SD Mean Difference (95% CI) from Baseline		Baseline Mean ± SD	45-Day Mean ± SD Mean Difference (95% CI) from Baseline	90-Day Mean ± SD Mean Difference (95% CI) from Baseline		
FBG (mg/dL)	95.82 ± 8.13	97.14 ± 6.37 1.32 (−10.75 to−1.89)	96.70 ± 8.40 0.88 (−10.37 to −1.40)	0.189	96.09 ± 15.78	99.26 ± 13.20 3.17 (−13.94 to 7.12)	101.50 ± 22.61 5.41 (−15.70 to 5.89)	0.646	0.133
BUN (mg/dL)	12.48 ± 2.51	10.85 ± 3.84 −1.63 (−3.50 to 16.04)	12.45 ± 2.80 −0.03 (−1.64 to 2.19)	0.381	12.43 ± 2.41	12.51 ± 2.36 0.08 (−4.25 to 16.79)	13.54 ± 2.89 1.11 (−1.64 to 2.19)	0.238	0.772
Creatinine (mg/dL)	0.70 ± 0.09	0.69 ± 0.11 −0.01 (−0.11 to 0.03)	0.66 ± 0.66 −0.04 (−0.09 to 0.09)	0.605	0.73 ± 0.14	0.68 ± 0.14 −0.05 (−0.11 to 0.03)	0.69 ± 0.15 −0.04 (−0.09 to 0.09)	0.473	0.972
AST (U/L)	22.71 ± 10.34	21.62 ± 8.47 −1.09 (−43.57 to 1.93)	23.00 ± 7.31 0.29 (−57.20 to3.18)	0.170	25.47 ± 13.96	25.37 ±11.48 0.12 (−43.12 to 1.49)	24.78 ± 7.22 −0.69 (−58.90 to 4.89)	0.480	0.193
ALT (U/L)	25.85 ± 418.66	26.95 ± 24.06 1.12 (−73.63 to −6.14)	28.00 ± 19.29 2.15 (−73.64 to −3.32)	0.531	28.72 ± 13.30	27.28 ± 13.06 −1.44 (−72.97 to −6.80)	30.76 ± 16.67 2.04 (−75.36 to −1.60)	0.278	0.142
ALP (U/L)	53.91 ± 6.28	56.70 ± 12.72 2.79 (−18.09 to 9.09)	56.81 ± 11.73 2.91 (−19.80 to 9.14)	0.279	62.80 ± 24.16	61.33± 13.67 −1.47 (−17.90 to 8.90)	60.19 ± 12.93 −2.61 (−20.25 to 9.59)	0.314	0.461
Sodium (mmol/L)	139.36 ± 0.92	138.61 ± 1.44 −0.75 (−0.97 to 1.09)	139.10 ± 1.64 −0.26 (−0.56 to 1.47)	0.308	140.54 ± 1.94	138.99 ± 1.92 −1.55 (−0.99 to 1.11)	139.57 ± 1.69 −0.97 (−0.56 to 1.47)	0.519	0.376
Potassium (mmol/L)	4.29 ± 0.40	4.32 ± 0.31 0.03 (−0.24 to 0.09)	4.15 ± 0.29 −0.14 (−0.31 to 0.06)	0.900	4.22 ± 0.30	4.25 ± 0.23 0.03 (−0.24 to 0.09)	4.16 ± 0.30 −0.06 (−0.31 to 0.06)	0.514	0.179
Chloride (mmol/L)	105.82 ± 1.99	103.91 ± 3.09 −1.91 (−0.94 to 2.06)	104.10 ± 2.84 −1.72 (−0.11 to 2.65)	0.166	105.54 ± 1.76	104.41 ± 1.50 −1.13 (−0.91 to 2.03)	105.29 ± 1.42 −0.25 (−0.15 to 2.68)	0.453	0.718
Hemoglobin (g/dL)	13.22 ± 1.357	13.03 ± 1.40 −0.19 (−0.64 to1.27)	13.32 ± 1.56 −0.1 (−1.13 to 0.84)	0.614	13.19 ± 1.26	13.08 ± 1.35 −0.11 (−0.63 to 1.26)	13.57 ± 1.70 0.38 (−1.14 to 0.85)	0.145	0.227
Hematocrit (%)	39.40 ± 4.147	39.55 ± 4.39 0.15 (−3.74 to 1.91)	39.85 ± 4.12 0.45 (−2.13 to 4.04)	0.939	42.00 ± 5.09	40.85 ± 5.12 −1.15 (−3.70 to 1.87)	40.84 ± 5.08 −1.16 (−2.17 to 4.08)	0.757	0.430
WBC counts (cell/cu.mm)	6395 ± 1775.70	7130 ± 2237.97 735 (−1689.10 to 865.08)	6955 ± 2711.86 560 (−238.86 to 2830.63)	0.566	7595 ± 1990.63	6965 ± 1967.97 −630 (−1669.13 to 845.11)	7236.84 ± 1990.87 −358.16 (−293.53 to 2885.30)	0.582	0.722
Neutrophil (%)	59.15 ± 5.76	60.6 ± 7.68 1.50 (−5.64 to 2.91)	60.25 ± 9.71 1.10 (−0.48 to 8.70)	0.585	62.40 ± 5.47	59.15 ± 7.07 −3.25 (−5.66 to 2.92)	60.37 ± 6.31 −2.03 (−0.46 to 8.68)	0.249	0.311
Lymphocyte (%)	36.45 ± 7.997	34.30 ± 7.97 −2.15 (−2.76 to 5.84)	34.45 ± 9.52 −2.01 (−8.81 to 0.25)	0.706	34.90 ± 5.73	35.2 ± 6.83 2.39 (−2.82 to 5.89)	33.84 ± 6.16 5.30 (−8.79 to 0.23)	0.232	0.592
Monocyte (%)	3.60 ± 1.095	3.15 ± 0.93 0.00 (−0.81 to 0.55)	3.35 ± 0.88 −0.25 (−0.63 to 0.97)	0.316	3.70 ± 1.13	3.65 ± 1.31 −0.17 (−0.80 to 0.55)	3.79 ± 1.23 0.74 (−0.64 to 0.98)	0.932	0.412
Eosinophil (%)	2.00 ± 0.00	2.00 ± 0.00 0.0 (-)	2.00 ± 0.00 0.0 (-)	1.000	2.00 ± 0.00	2.00 ± 0.00 0.0 (-)	2 ± 0.00 0 (-)	1.000	1.000
Platelet counts (cell/cu.mm)	266,575 ± 89,903.64	290,050 ± 70,576.93 23,475 (−61,352.59 to 12,536.85)	279,900 ± 87,735.19 13,325 (−13,678.20 to 71,760.45)	0.647	263,040 ± 919,78.48	279,130 ± 92,468.04 16,090 (−60,706.21 to 11,890.48)	286,947 ± 63,605.97 23,907 (−14,090.77 to 72,173.02)	0.648	0.430
MCV (fL)	80.44 ± 6.988	80.62 ± 7.02 0.17 (−0.50 to 7.77)	80.46 ± 5.65 0.02 (−9.58 to 0.35)	0.996	78.39 ± 8.99	79.77 ± 7.37 1.38 (−0.51 to 7.78)	76.31 ± 8.52 −2.90 (−9.63 to 0.40)	0.716	0.510
MCH (pg)	26.74 ± 2.93	26.34 ± 3.27 −0.40 (−0.55 to 3.55)	26.96 ± 3.07 0.22 (−34.92 to 9.26)	0.798	26.11 ± 3.36	25.07 ± 2.92 −1.04 (−0.55 to 3.55)	25.58 ± 4.16 −0.53 (−35.03 to 9.37)	0.573	0.498
MCHC (g/dL)	33.53 ± 1.139	32.74 ± 1.66 −0.79 (−0.13 to 1.91)	33.48 ± 1.86 −0.05 (−2.03 to 0.02)	0.877	32.14 ± 1.45	31.08 ± 1.37 −1.06 (−0.13 to 1.91)	33.39 ± 2.27 1.25 (−2.03 to 0.03)	0.270	0.913

Data expressed as mean ± standard deviations. FBG; Fasting blood glucose, BUN; Blood Urea Nitrogen, ALT; Alanine aminotransferase, AST; Aspartate aminotransferase, ALP; Alkaline phosphatase, WBC; white blood cell, MCV; mean corpuscular volume, MCH; mean corpuscular hemoglobin, MCHC; mean corpuscular hemoglobin concentration.

**Table 3:** Effect of *L. paracasei* TISTR 2593 supplementation on blood lipid profiles.

Lipid Profiles	*L. paracasei* TISTR 2593			Placebo			*p*-Value between Groups
	Baseline Mean ± SD	45-Day Mean ± SD Mean Difference (95% CI) from Baseline	90-Day Mean ± SD Mean Difference (95% CI) from Baseline	Baseline Mean ± SD	45-Day Mean ± SD Mean Difference (95% CI) from Baseline	90-Day Mean ± SD Mean Difference (95% CI) from Baseline	
Total cholesterol (mg/dL)	233.50 ± 41.59	224.90 ± 34.41 −8.60 (−27.68 to 21.98)	227.75 ± 33.45 −5.75 (−36.28 to 1 3.38)	231.48 ± 40.51	252.13 ± 47.46 20.65 (−46.01 to 4.71)	246.70 ± 40.42 15.22 (−41.52 to 11.07)	0.072
Triglyceride (mg/dL)	143.50 ± 40.05	153.60 ± 44.90 10.10 (−32.90 to 38.37)	151.21 ± 41.42 7.71 (−23.17 to 48.10)	146.09 ± 55.74	157.96 ± 61.24 11.87 (−47.99 to 24.25)	157.14 ± 66.99 11.06 (−48.03 to 25.92)	0.080
HDL-C (mg/dL)	53.35 ± 10.71	56.75 ± 8.98 3.40 (−7.27 to 9.07)	55.85 ± 10.83 2.50 (−3.87 to 12.47)	52.48 ± 10.65	58.65 ± 14.95 6.17 (−13.46 to 1.11)	57.00 ± 10.90 4.52 (−11.98 to 2.94)	0.125
LDL-C (mg/dL)	155.15 ± 33.03	137.40 ± 32.44 * −17.75 (−23.84 to 18.94)	137.63 ± 29.24 * −17.52 (−41.59 to 1.19)	151.61 ± 36.13	161.87 ± 36.98 10.26 (−32.39 to 11.87)	163.00 ± 39.94 11.39 (−35.01 to 12.23)	0.004 ^+^

Data expressed as mean ± standard deviations. * Significant difference from baseline within each group (*p* < 0.05). ^+^ Significant difference between groups (*p* < 0.05). HDL-C; High-Density Lipoprotein Cholesterol, LDL-C; Low- Density Lipoprotein Cholesterol.

**Table 4:** Effect of *L. paracasei* TISTR 2593 supplement on adiponectin, apolipoprotein E, and total bile acid level.

Parameters	*L. paracasei* TISTR 2593			Placebo			*p*-Value between Groups
	Baseline Mean ± SD	45-Day Mean ± SD Mean Difference (95% CI) from Baseline	90-Day Mean ± SD Mean Difference (95% CI) from Baseline	Baseline Mean ± SD	45-Day Mean ± SD Mean Difference (95% CI) from Baseline	90-Day Mean ± SD Mean Difference (95% CI) from Baseline	
MDA (nmol/mL)	0.069 ± 0.04	0.045 ± 0.02 * −0.03 (0.01 to 0.04)	0.030 ± 0.01 *** −0.04 (0.02 to 0.06)	0.071 ± 0.03	0.064 ± 0.01 −0.01 (−0.07 to −0.02)	0.078 ± 0.01 0.01 (−0.02 to 0.06)	0.000 ^+++^
GSH (µg/mL)	3.521 ± 1.94	4.031 ± 1.33 0.51 (−1.40 to 0.39)	3.387 ± 0.53 −0.14 (−0.78 to 1.05)	3.561 ± 1.46	3.844 ± 1.93 0.28 (−1.21 to 0.64)	3.337 ± 0.46 −0.23 (−0.70 to 1.15)	0.150
TNF-α (pg/mL)	11.163 ± 6.87	9.656 ± 1.74 −1.51 (−1.61 to 4.62)	6.063 ± 2.79 ** −5.10 (1.90 to 8.30)	11.541 ± 7.39	12.882 ± 2.57 1.34 (−4.37 to 1.81)	9.759 ± 4.50 −1.78 (−1.29 to 4.98)	0.024 ^+^
IL-6 (pg/mL)	1.207 ± 0.39	1.186 ± 0.21 −0.02 (−0.15 to 0.19)	1.052 ± 0.11 −0.16 (0.01 to 0.33)	1.325 ± 0.94	1.463 ± 0.43 0.14 (−0.54 to 0.27)	1.218 ± 0.29 −0.11 (−0.22 to 0.54)	0.134
IL-10 (pg/mL)	4.361 ± 2.52	5.138 ± 3.71 0.78 (−2.68 to 1.12)	3.763 ± 2.03 −0.60 (−1.46 to 2.66)	4.357 ± 2.55	4.214 ± 4.03 −0.14 (−1.98 to 2.27)	3.877 ± 2.364 −0.480 (−1.08 to 2.84)	0.659
MCP-1 (pg/mL)	1.942 ± 1.19	1.788 ± 1.22 −0.10 (−0.60 to 0.80)	1.800 ± 1.01−0.09 (−0.65 to 0.83)	1.890 ± 1.94	1.823 ± 1.06 −0.12 (−0.58 to 0.81)	2.037 ± 1.37 0.10 (−0.81 to 0.62)	0.360
Adiponectin (ng/mL)	37.199 ± 0.57	35.132 ± 0.60 *** −2.07 (1.45 to 2.68)	35.251 ± 1.47 *** −3.95 (3.33 to 4.56)	37.523 ± 0.44	32.627 ± 0.55 *** −1.90 (1.36 to 2.43)	32.768 ± 1.96 *** −5.76 (5.20 to 6.31)	0.005 ^++^
APOE (ng/mL)	0.751 ± 0.09	0.872 ± 0.16 * 0.12 (−0.21 to −0.03)	0.887 ± 0.12 ** 0.14 (−0.23 to −0.04)	0.783 ± 0.09	0.817 ± 0.10 0.03 (−0.10 to 0.03)	0.806 ± 0.06 0.02 (−0.09 to 0.04)	0.088
TBA (µmol/L)	4.560 ± 3.13	4.214 ± 3.03 −0.35 (−2.24 to 2.93)	5.715 ± 4.89 1.16 (−3.66 to 1.35)	4.619 ± 3.02	5.701 ± 6.15 1.08 (−4.41 to 2.25)	5.983 ± 5.04 1.36 (−4.70 to 1.96)	0.100

Data expressed as mean ± standard deviations. *, **, *** Significant difference from baseline within each group (*p* < 0.05, 0.01 and 0.001, respectively). ^+^, ^++^, ^+++^ Significant difference between groups (*p* < 0.05, 0.01, and 0.001, respectively). MDA; Malondialdehyde, GSH; Glutathione, TNF- α; Tumor necrosis factor alpha, IL−6; Interleukin 6; Interleukin 10, APOE; Apolipoprotein E, TBA; total bile acid.

## Data Availability

The data supporting the findings of this study are included in this article.
