# Correction: Khongrum et al. Safety and Effects of *Lactobacillus paracasei* TISTR 2593 Supplementation on Improving Cholesterol Metabolism and Atherosclerosis-Related Parameters in Subjects with Hypercholesterolemia: A Randomized, Double-Blind, Placebo-Controlled Clinical Trial. *Nutrients* 2023, *15*, 661

**DOI:** 10.3390/nu16152521

**Published:** 2024-08-02

**Authors:** Jurairat Khongrum, Pratoomporn Yingthongchai, Kongsak Boonyapranai, Wachira Wongtanasarasin, Paitoon Aobchey, Suriya Tateing, Aree Prachansuwan, Jaruwan Sitdhipol, Kanidta Niwasabutra, Punnathorn Thaveethaptaikul, Pongsathon Phapugrangkul, Pennapa Chonpathompikunlert

**Affiliations:** 1 Science and Technology Research Institute, Chiang Mai University, Chiang Mai 50200, Thailand; pratoomporn.y@cmu.ac.th (P.Y.); paitoon.aob@cmu.ac.th (P.A.); 2 Functional Food Research Center for Well-Being, Science and Technology Research Institute, Chiang Mai University, Chiang Mai 50200, Thailand; 3 Research Institute for Health Science, Chiang Mai University, Chiang Mai 50200, Thailand; kongsak.b@cmu.ac.th; 4 Department of Emergency Medicine, Faculty of Medicine, Chiang Mai University, Chiang Mai 50200, Thailand; wachira.wong@cmu.ac.th; 5 Department of Plant and Soil Sciences, Faculty of Agriculture, Chiang Mai University, Chiang Mai 50200, Thailand; suriya.t@cmu.ac.th; 6 Institute of Nutrition, Mahidol University, Nakhon Pathom 73170, Thailand; aree.prc@mahidol.ac.th; 7 Biodiversity Research Centre (BRC), Thailand Institute of Scientific and Technological Research (TISTR), Pathum Thani 12120, Thailand; jaruwan_s@tistr.or.th (J.S.); kanidta@tistr.or.th (K.N.); punnathorn@tistr.or.th (P.T.); pongsaton@tistr.or.th (P.P.)

Error in Table

In the original publication [1], there was a mistake in Table 2 as published. In the table, we quoted the baseline of blood parameter-ALT of the *L. paracasei* TISTR 2593 group which was at “25.85 ± 418.66 U/L”.

The corrected blood parameter appears below.

ALT on the baseline of the *L. paracasei* TISTR 2593 group was “25.85 ± 18.66 U/L”.

Text Correction

There was an error in the original publication in Materials and Methods and Discussion [1]. We stated that the dose of “*L. paracasei* TISTR 2593” was at “1.05 × 10^10^ CFU/g”.

A correction has been made to Materials and Methods, Section 2.2., paragraph 2.

The correct dose of *L. paracasei* TISTR 2593 is “1.05 × 10^9^ CFU/g”.

A correction has been made to Discussion, paragraphs 1 and 2.

The correct dose of *L. paracasei* TISTR 2593 is “1.05 × 10^9^ CFU/g”.

## References

[1] Khongrum J., Yingthongchai P., Boonyapranai K., Wongtanasarasin W., Aobchey P., Tateing S., Prachansuwan A., Sitdhipol J., Niwasabutra K., Thaveethaptaikul P. (2023). Safety and Effects of *Lactobacillus paracasei* TISTR 2593 Supplementation on Improving Cholesterol Metabolism and Atherosclerosis-Related Parameters in Subjects with Hypercholesterolemia: A Randomized, Double-Blind, Placebo-Controlled Clinical Trial. Nutrients.

